# Effects of 3% binahong ( *Anredera cordifolia*) leaf extract gel on alveolar bone healing in post-extraction tooth socket wound in Wistar rats ( *Rattus norvegicus*)

**DOI:** 10.12688/f1000research.72982.2

**Published:** 2022-04-05

**Authors:** Olivia Avriyanti Hanafiah, Diana Sofia Hanafiah, Gostry Aldica Dohude, Denny Satria, Livita Livita, Nindha Siti Moudy, Rahma Rahma

**Affiliations:** 1 Department of Oral and Maxillofacial Surgery, Faculty of Dentistry, Universitas Sumatera Utara, Medan, 20155, Indonesia; 2 Department of Agroecotechnology, Faculty of Agriculture, Universitas Sumatera Utara, Medan, 20155, Indonesia; 3 Department of Pharmaceutical Biology, Faculty of Pharmacy, Universitas Sumatera Utara, Medan, 20155, Indonesia; 4 Faculty of Dentistry, Universitas Sumatera Utara, Medan, 20155, Indonesia

**Keywords:** Anredera cordifolia, tooth extraction, wound healing, fibroblasts, osteoblasts, osteocytes

## Abstract

**Background:** Binahong ( *Anredera cordifolia* (Ten.) STEENIS) is a widely available herbal plant in Indonesia and has been intensely researched for its healing abilities due to its biological activities, but few have studied its capability in accelerating hard tissue healing in post-extraction tooth sockets. The purpose of this study was to analyse the effects of 3% binahong leaf extract gel on alveolar bone healing in post-extraction sockets in Wistar rats.

**Methods:** In this study, 48 male Wistar rats were randomly allocated to twelve groups. After the extraction of the left mandibular incisor, sockets in Group I to IV were given 3% binahong leaf extract gel, group V to VIII were given a control gel, and group IX to XII were given Gengigel ^®^ for 14 days. The residual socket volume (RSV) and fibroblast proliferation were observed on the 3 ^rd^, 7 ^th^, and 14 ^th^ day post-extraction, while the osteoblast and osteocyte proliferation were observed on the 7 ^th^, 14 ^th^, and 28 ^th^ day post-extraction. The RSV data were analysed using repeated measure ANOVA and one-way ANOVA, while the histopathological data were analysed using one-way ANOVA.

**Results:** The results showed that the binahong group had the lowest RSV and the highest fibroblast proliferation compared to the other groups on the 7th day (p<0.05) and the highest osteoblast and osteocyte proliferation compared to the other groups on the 14 ^th^ day (p<0.05).

**Conclusion:** The experiment showed that 3% binahong leaf extract gel could accelerate wound closure, which was characterized by a greater decrease in the RSV value in comparison to the other treatment groups and could enhance alveolar bone healing by increasing the proliferation of fibroblasts, osteoblasts, and osteocytes.

## Introduction

Tooth extraction will leave a socket wound and can affect a person's quality of life, especially in terms of the ability to eat and speak. ^1^^,^^2^ Socket wound healing involves the healing of soft tissue that is, comprised of the gingiva and connective tissue, as well as the healing of hard tissue, the alveolar bone. ^3^ Both soft tissue and hard tissue undergo the same healing phases, namely the inflammatory, proliferative, and remodelling phases. ^2^

Some of the cells that play important roles during the healing of socket wounds are fibroblasts, osteoblasts, and osteocytes. At the beginning of alveolar bone healing, the cells that are more active are fibroblasts because these cells produce collagen, glycosaminoglycans, proteoglycans, and adhesive glycoproteins that will form granulation tissue. ^4^ Granulation tissue is rich in blood vessels and aims to restore tissue unity and prepare for bone formation by osteoblasts, osteoclasts, and osteocytes in the remodelling phase. ^5^^,^^6^ In mice, the fibroblasts in alveolar bone begin to proliferate on the 3 ^rd^ day after tooth extraction and continue to increase until the 7 ^th^ day. ^7^^,^^8^ From the 7 ^th^ day to day 14 ^th^ day, there will be a decrease in the number of fibroblasts as new alveolar bone starts to fill the socket. ^8^ A proper proliferation of fibroblasts will trigger good bone formation and accelerate socket wound closure because fibroblasts also take part in wound retraction. ^9^ Osteoblasts are mononuclear cells that synthesize osteoid matrix and play an important role from the proliferative phase to the remodelling phase of healing. ^2^^,^^3^^,^^10^^,^^11^ Based on previous research, osteoblasts in the bone healing process after tooth extraction can be found on the 7 ^th^ day and will continue to differentiate for more than 21 days. ^3^^,^^7^ Osteocytes are the matured form of osteoblasts. ^12^ When osteoblasts form bone, they can be trapped in the matrix formed by osteoblasts and differentiate into osteocytes. ^11^^,^^12^ In the bone healing process, osteocytes play a role in bone remodelling by maintaining the integrity and vitality of the new bone. ^11^ Based on previous research, there are more osteocytes on the 28 ^th^ day post-extraction. ^13^

Currently, alternative medicine with herbal ingredients has started to be used to help wound healing. The employment of natural active ingredients in herbal medicine can also reduce the cost of wound care. ^14^ Several plants that have previously been investigated for their effects on wound healing are Mobe ( *Artocarpus lakoocha*), which can increase cell proliferation and migration; Miswak ( *Salvadora persica*), which can increase the formation of alveolar bone; and Moringa ( *Moringa oleifera*), which can increase the expression of bone-forming proteins. ^15^^–^^17^ Another plant, namely *Aloe vera*, has even been proven effective in accelerating the socket wound healing after tooth extraction in humans. ^18^

Binahong ( *Anredera cordifolia* (Ten.) STEENIS) is a plant used in herbal medicine. Binahong is often considered as a wild plant and harmful to other plants because its growth is invasive. However, the characteristics of Binahong, which are its abilities to grow easily and rapidly in varied climates, can also be advantages because Binahong can be used as an alternative medicine that is also highly-sustainable. ^19^^,^^20^ Binahong is known to have pharmacological properties, such as antiinflammatory, antibacterial, antifungal, antiviral, and antioxidant properties. ^19^^,^^21^ It could also improve kidney function, counter hyperlipidemia, reduce hypertension, become anticancer, act as an analgesic, and help wound healing, including tooth socket wounds. ^19^^,^^21^ Binahong also has been used in traditional medicine by placing its leaves over wounds and it has shown an affinity in accelerating wound healing. ^21^^,^^22^ This is due to the presence of the secondary metabolites contained in the Binahong leaf extract such as tannins, saponins, flavonoids, alkaloids, anthraquinones, phenolic acids, triterpenes, steroids, and glycosides. ^23^ The flavonoids found are quercetin, rutin, apigenin, apigetrin, morin, myricetin, and vitexin. ^24^^–^^27^ The phenolic acid found in Binahong leaf extract is P-coumaric acid, and the triterpenes found are ursolic acid and oleanolic acid. ^24^^,^^28^ A few studies have been conducted to observe the effects of Binahong leaf extract on wound healing. Hanafiah *et al* showed that Binahong leaf extract could increase the proliferation of NIH-3T3 fibroblasts and also proved that 3% Binahong leaf extract gel could accelerate palatal mucosal wound healing in rats by increasing the proliferation of fibroblasts. ^29^^,^^30^ The study of Khoswanto *et al* showed that the application of 10% Binahong leaf extract gel could increase the expression of BMP-2 (bone morphogenetic protein-2) and osteoblasts in the tooth socket wound of rats on the 7 ^th^ day post-extraction. ^3^

The purpose of this study was to determine the effects of 3% Binahong leaf extract gel application on the acceleration of socket wound closure and the proliferation of fibroblasts, osteoblasts, and osteocytes in the healing of alveolar bone in tooth socket wounds up to 28 days post-extraction.

## Methods

### Ethical considerations

All experimental procedures in this study were carried out in accordance with the Institutional Animal Care and Usage Committee (ARRIVE) guidelines 2.0. Ethical clearance had been approved by the Health Research Ethics Committee (KEPK) at the Department of Biology, Faculty of Mathematics and Natural Sciences, Universitas Sumatera Utara, Medan, North Sumatra, Indonesia with letter numbers: 0206/KEPH-FMIPA/2021 and 0202/KEPH-FMIPA/2021.

### Animals

*Sample size*

The sample size in this study was calculated based on previous research of a similar nature. ^3^ The effect size (Cohen’s d) between the experimental group and the control group from the previous research was 3.31. ^3^ The minimum sample size obtained from the calculations was three animals and the final sample size was adjusted to four animals per group (with a total of 12 groups) or 48 animals in total. There was a 10% addition from the minimum sample size to anticipate sample exclusion during the experiment.

*Rats*

Animals used in this study were 48 male Wistar rats ( *Rattus norvegicus*) of two to three months old and with an average body weight of 200-250 grams. The rats should also be in good health and had never received any research treatment previously. Rats with abnormalities or those that died before the experiment ended were excluded from the study. The rats that died were not replaced as long as the minimum sample size was still maintained. The rats were acquired and housed at the Animal House in the Faculty of Mathematics and Natural Sciences in Universitas Sumatera Utara, Medan, North Sumatra, Indonesia. Before administering the treatment, the rats were acclimatized for seven days to assure that the rats could adapt to their surroundings and were allowed *ad libitum* feeding and drinking.

### Study design and groups

**Figure 1.**
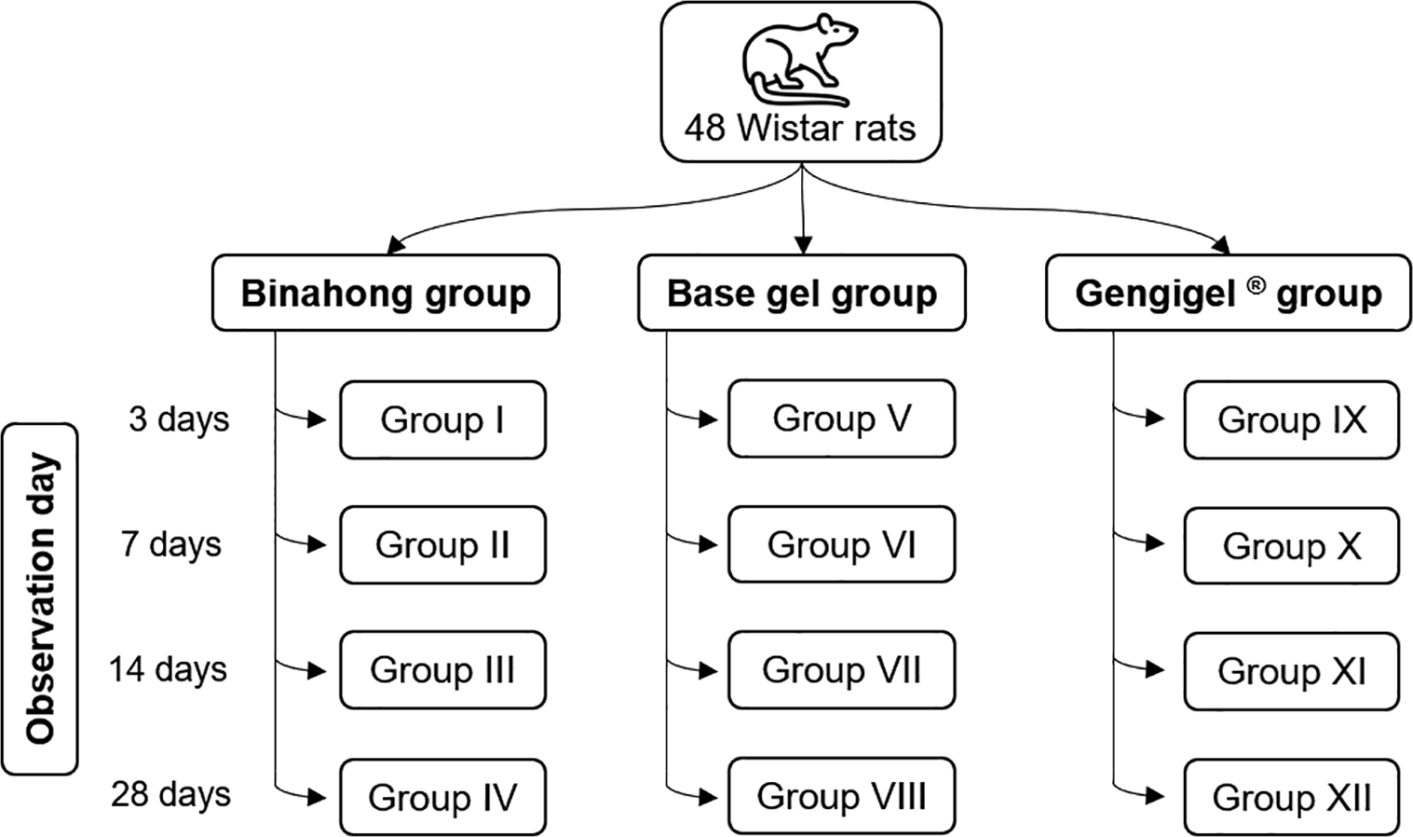
Group allocation; each group is differentiated from one another based on its treatment and observation day and is comprised of four Wistar rats.

The study conducted was an *in vivo* experiment with a post-test only control design. The rats were randomized by simple random sampling and allocated into twelve groups by the Animal House lab technicians: group I to IV were given 3% Binahong leaf extract gel, group V to VIII were given base gel as the negative control, and group IX to XII were given Gengigel ^®^ as the positive control ( Figure 1). The treatments were given for 14 days as that was the approximate amount of time needed for the socket wound to close completely. ^2^ The residual socket volume (RSV) and fibroblast proliferation were observed on the 3 ^rd^, 7 ^th^, and 14 ^th^ day post-extraction, while the osteoblast and osteocyte proliferation (cells) were observed on the 7 ^th^, 14 ^th^, and 28 ^th^ day post-extraction. All researchers were blinded to the box assignation of the rats, except for the researchers who were in charge of applying the gel to the socket wounds.

### Tooth extraction

Wistar rats were anesthetized intraperitoneally using a combination of 91 mg/kgBW ketamine (Agrovet market, Peru) and 9.1 mg/kgBW xylazine (Interchemie, Netherlands) with an anesthetic dose of 0.1 mL/100 g rat weight to alleviate the pain induced by the procedure. ^31^ After that, the mandibular left incisor was extracted using an artery clamp (Wells Spencer, London) with a luxation movement. ^32^ After the extraction, the socket was irrigated with distilled water to clean the socket from debris. The extraction was performed by the same veterinarian who was blinded to the group allocation.

### Binahong leaf extract gel and base gel preparation

The Binahong leaf extract used in this study was obtained from the Pharmacognosy Laboratory, Faculty of Pharmacy, Universitas Sumatera Utara, Medan, North Sumatra, Indonesia. A total of 400 g fully opened Binahong leaves and aged approximately 12 weeks were selected and obtained from Simpang Perdagangan village, Tigabinanga district, Karo regency, North Sumatra, Indonesia in April 2017. Tigabinanga district is located at coordinate 3.0729, 98.2415. It has an altitude of 490 to 750 meter above sea level. ^33^ Karo regency has a tropical climate, with rainy season (August-January, March-May) and dry season (February, June, July). ^34^ Binahong leaves were extracted by the maceration method using 80% ethanol (Smart Lab Indonesia, Indonesia) as the solvent. The Binahong leaves were initially grinded into powder with an electric blender (Philips HR2115, Indonesia) and then soaked in 80% ethanol in a closed container and distilled for five days at room temperature. After five days, the 80% ethanol solvent was replaced with new solvent and soaked again for two days. Subsequently, a final filtering process was carried out. Then, a water bath was used to evaporate the solvent until the extract dried. ^35^

**Figure 2.**
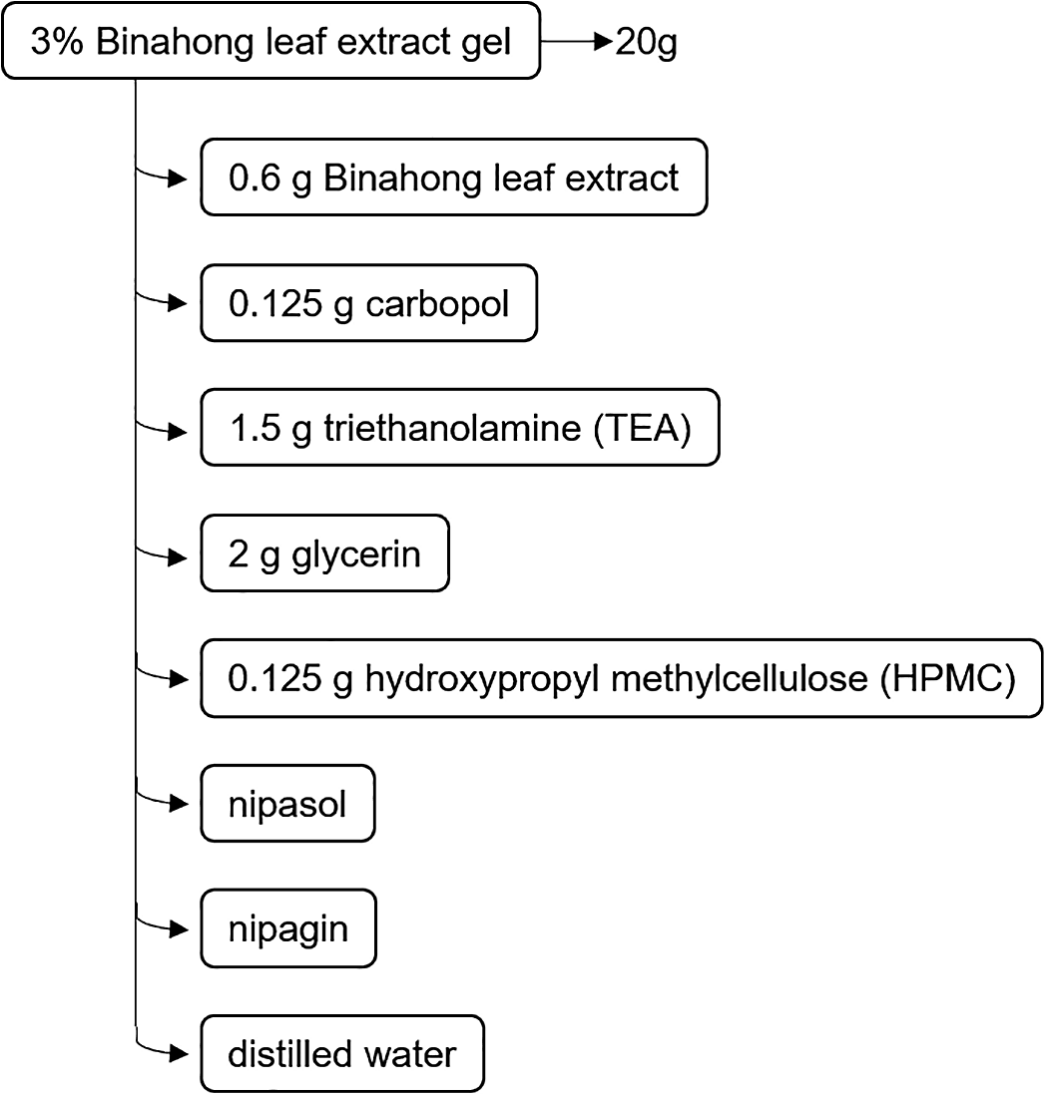
The composition of 3% Binahong leaf extract gel.

The base gel was made by adding 10 mL of hot distilled water to a mortar, then 0.125 g of carbopol (Merck, Germany) was added and the mixture was stirred with a pestle. Next, 1.5 g of triethanolamine (TEA) (Merck, Germany) and 2 g of glycerin (Merck, Germany) were added and the mixture was stirred until it was homogeneous. In a second mortar, a mixture of 10 mL distilled water, 0.125 g of hydroxypropyl methylcellulose (HPMC) (Merck, Germany), nipagin (Merck, Germany), and nipasol (Merck, Germany) were stirred until homogeneous. ^30^ The second mortar mixture was poured into the first mortar and the mixture was stirred until it was homogeneous. To obtain 20 g of 3% Binahong leaf extract gel, 0.6 g of Binahong leaf extract was added to the base gel and the mixture was stirred until it was homogenous ( Figure 2). The gel was reproduced every 3 to 4 days to ensure its freshness.

### Application of gels

The socket wounds in group I to VI were applied with 3% Binahong leaf extract gel; group V to VIII were given base gel; and group IX to XII were given Gengigel ^®^ (Ricerfarma, Italy). In every application, 0.1 mL of gel was applied directly to the socket wound using a 1 mL syringe (One Med Health Care, Indonesia) with a bent needle irrigation tip (Ivoclar Vivadent, ∅: 1,2 mm, Liechtenstein) until the gel covered the entire wound surface to make sure that the gel would be directly in contact with the wound. Applications were made twice a day in the morning at 8.00 a.m.-10.00 a.m. and in the afternoon at 4.00 p.m.-6.00 p.m. for 14 days. The time was chosen to ensure consistent schedule for the gel application each day and the application was conducted by a researcher who was aware of the group allocation.

### Clinical measurement of residual socket volume

Measurements of the socket volume were made with a digital caliper (Digital Caliper, China *)*, a pair of compasses (Joyko ^®^, Indonesia), and a periodontal probe (Kohler, NR-3182, Germany) and were performed on the rats in group III, VII, XI, so that repeated measurements could be made on the same samples. The measurements were performed by the same researcher blinded. The average socket volume was calculated for each male Wistar rat. To determine the residual socket volume for each rat, the socket volume obtained from the measurements on the 3 ^rd^ day, 7 ^th^ day, and 14 ^th^ day were each divided by the socket volume on the 1 ^st^ day. Socket volume = mesiodistal width × buccolingual width × probing depth. ^36^

### Histopathological analysis

After reaching the 3 ^rd^ day (group I, V, IX), the 7 ^th^ day (group II, VI, X), the 14 ^th^ day (group III, VII, XI), and the 28 ^th^ day (group IV, VIII, XII) post-extraction, the rats from the respective groups were sacrificed by cervical dislocation. This method was chosen to ensure rapid termination and lower chance of tissue contamination. The mandible of the rats was removed from the skull and the socket wound tissue was excised. Fresh tissue was fixed in 10% Buffered Neutral Formalin (BNF) solution (Milestone Medical, Italy) with a pH of 6.8-7.0 and the ratio of tissue to the BNF solution was 1:10. ^37^ The tissue containers were labelled and fixation was carried out for 12-48 hours, after which the tissue was immersed in 10% EDTA solution (Merck, Germany) for 10 days for decalcification. ^38^ The tissue was then cut using a scalpel with a thickness of 4 mm and the tissue was placed in tissue cassettes and put into a basket. ^37^ The baskets were loaded into an automatic processor machine and then transferred to a dehydration machine for tissue dehydration. ^37^ The next stage was embedding, in which the tissue was placed in a mould and submerged in liquid paraffin. ^37^ The cooled paraffin blocks were cut with a thickness of 4-5 μm using a microtome machine and the slices were placed in a water bath at 45°C. ^37^ The tissue slices would be collected on clean glass slides. The slides were labelled and placed in an incubator at 37°C to dry overnight. ^37^ The slides were then stained with haematoxylin-eosin (Merck, Germany) to see osteoblasts and osteocytes and with Masson's Trichrome (Merck, Germany) to see fibroblasts microscopically. ^37^ The stained tissue slides were then observed under an electric microscope (Primo Star, Carl Zeiss, Germany) with a magnification of 400 times in 10 viewing fields. Fibroblasts when observed under microscope are spindle-shaped with an ovoid nucleus and are commonly found in connective tissue. ^4^ Osteoblasts when active are spindle-shaped or cuboidal, large and broad with abundant basophilic cytoplasm. When inactive, osteoblasts are flat, spindle-shaped, and lined up along the bone matrix surface. ^10^^,^^11^^,^^39^ Meanwhile, osteocytes are smaller than osteoblasts, branched out, and are trapped in lacunae among the matrix formed by osteoblasts. ^11^^,^^39^

The calculation of the mean cell count was carried out by two observers blinded to avoid bias and was aided with a tally counter (SXH, 5136, China) and a calculator (Casio, ƒχ-82MS, Japan). The results of the fibroblast, osteoblast, and osteocyte cell counts from 10 viewing fields were averaged. ^40^

### Statistical analysis

The data were first analysed with Shapiro-Wilk normality test to ascertain the distribution of the data. Data that were normally distributed would be analysed with parametric tests. The RSV data were analysed using repeated measures ANOVA and one-way ANOVA test, while the histopathological data of the mean cell count of fibroblasts, osteoblasts, and osteocytes were analysed using one-way ANOVA test. The multiple comparison tests were performed with Least Significant Difference (LSD) test. The results were considered as significant if the p-value was below 0.05. Statistical analysis was performed using the Statistical Package for the Social Sciences (SPSS), version 21 (IBM ^®^ Inc., USA) by a researcher who was aware of the group allocation.

## Results

### Clinical measurement of residual socket volume

**Figure 3.**
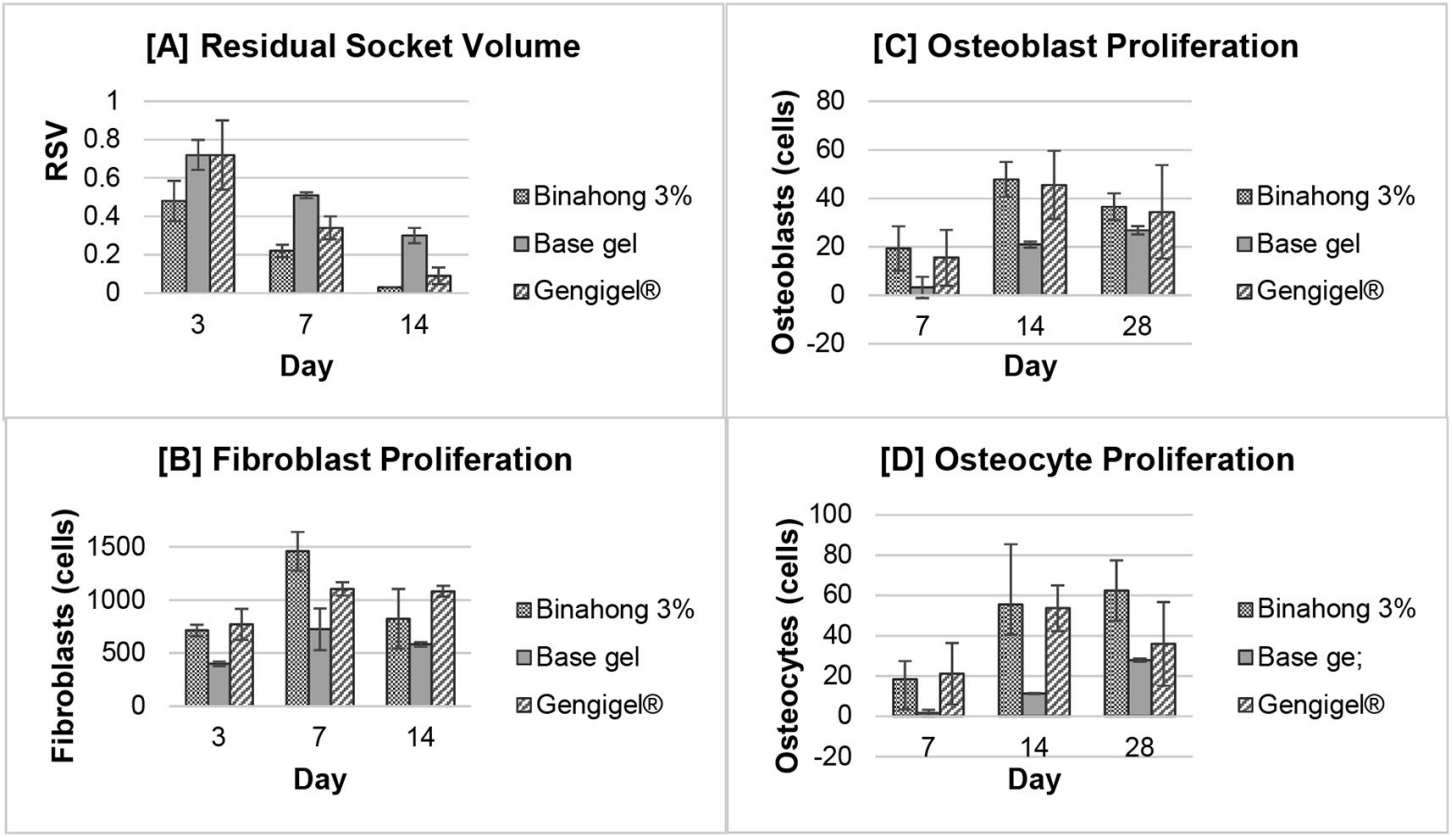
Clinical and histopathological analysis of alveolar bone healing between different treatment groups. Results were shown as the means of the residual socket volume (clinical observation) and the number of fibroblast, osteoblast, and osteocyte cells (histopathological observation).

The residual socket volume (RSV) was measured on the 3 ^rd^, 7 ^th^, and 14 ^th^ day post-extraction. The RSV in all treatment groups decreased over time and based on the repeated measures ANOVA test, there were significant differences in the RSV on the 3 ^rd^, 7 ^th^, and 14 ^th^ day in the Binahong group, the Gengigel ^®^ group, and the base gel group (p < 0.05) ( Figure 3A). In the Binahong group, significant RSV differences were observed between the 3 ^rd^ and 7 ^th^ day, between the 3 ^rd^ and 14 ^th^ day, and between the 7 ^th^ and 14 ^th^ day, while in the Gengigel ^®^ group and the base gel group, significant RSV differences were only observed between the 3 ^rd^ and 14 ^th^ and 7 ^th^ and 14 ^th^ day (p < 0.05). There was no difference in the RSV between the 3 ^rd^ and 7 ^th^ day in the Gengigel ^®^ group and the base gel group ( Table 1).

**Table 1.** Effects of each treatment on socket wounds for different time periods.

Treatment	Observation (day)	RSV (Mean ± SD)	Fibroblast (cells) (Mean ± SD)	Observation (day)	Osteoblast (cells) (Mean ± SD)	Osteocyte (cells) (Mean ± SD)
**Binahong 3%**	**3**	0.48 ± 0.105 ^a^	713.40 ± 54.896 ^a^	**7**	19.33 ± 9.129 ^a^	18.38 ± 9.068 ^a^
	**7**	0.22 ± 0.032 ^b^	1458.95 ± 182.654 ^b^	**14**	47.78 ± 7.208 ^b^	55.50 ± 29.928 ^b^
	**14**	0.03 ± 0.000 ^c^	824.27 ± 280.527 ^a^	**28**	36.49 ± 5.545 ^b^	62.44 ± 14.987 ^b^
**Base gel**	**3**	0.72 ± 0.078 ^a^	399.85 ± 20.977 ^a^	**7**	3.25 ± 4.356 ^a^	1.60 ± 1.709 ^a^
	**7**	0.51 ± 0.015 ^a^	724.87 ± 196.289 ^b^	**14**	20.88 ± 1.239 ^b^	11.37 ± 0.252 ^b^
	**14**	0.30 ± 0.040 ^b^	582.07 ± 22.830	**28**	26.78 ± 1.728 ^c^	27.93 ± 0.861 ^c^
**Gengigel ^®^**	**3**	0.72 ± 0.180 ^a^	771.70 ± 146.452 ^a^	**7**	15.48 ± 11.487	21.18 ± 15.229
	**7**	0.34 ± 0.060 ^a^	1105.40 ± 62.366 ^b^	**14**	45.52 ± 14.056	53.63 ± 11.388
	**14**	0.09 ± 0.044 ^b^	1082.87 ± 50.217 ^b^	**28**	34.42 ± 19.269	35.93 ± 20.740

The parameters examined to show the effects of each treatment were the residual socket value (RSV) in the clinical observation and the number of fibroblasts, osteoblasts, and osteocytes in the histopathological observation. The values shown were the mean ± standard deviation of at least three replicates. Different alphabets indicated significant statistical differences (p < 0.05) between the time periods analysed with the LSD test. The lack of alphabets or the same alphabets indicated no statistical differences (p > 0.05) between the time periods analysed with the LSD test.

Based on the one-way ANOVA test, there was a significant difference in the RSV among the treatment groups on the 7 ^th^ and 14 ^th^ day (p < 0.05). On the 7 ^th^ day, there were significant RSV differences between the Binahong group and the Gengigel ^®^ group, between the Binahong group and the base gel group, and between the Gengigel ^®^ group and the base gel group, while on the 14 ^th^ day, the significant RSV differences were only between the Binahong group and the base gel group and between the Gengigel ^®^ group and the base gel group ( Table 2).

**Table 2.** Comparison of the effects of different treatments on socket wounds.

Observation (day)	Treatment	RSV (Mean ± SD)	Fibroblast (cells) (Mean ± SD)	Observation (day)	Osteoblast (cells) (Mean ± SD)	Osteocyte (cells) (Mean ± SD)
**3**	**Binahong 3%**	**0.48 ± 0.105**	**713.40 ± 54.896** ^**a**^	**7**	19.33 ± 9.129	18.38 ± 9.068
	**Base gel**	**0.72 ± 0.078**	**399.85 ± 20.977** ^**b**^		3.25 ± 4.356	1.60 ± 1.709
	**Gengigel ^®^**	**0.72 ± 0.180**	**771.70 ± 146.452** ^**a**^		15.48 ± 11.487	21.18 ± 15.229
**7**	**Binahong 3%**	**0.22 ± 0.032** ^**a**^	**1458.95 ± 182.654** ^**a**^	**14**	47.78 ± 7.208 ^a^	55.50 ± 29.928 ^a^
	**Base gel**	**0.51 ± 0.015** ^**b**^	**724.87 ± 196.289** ^**b**^		20.88 ± 1.239 ^b^	11.37 ± 0.252 ^b^
	**Gengigel ^®^**	**0.34 ± 0.060** ^**c**^	**1105.40 ± 62.366** ^**c**^		45.52 ± 14.056 ^a^	53.63 ± 11.388 ^a^
**14**	**Binahong 3%**	**0.03 ± 0.000** ^**a**^	**824.27 ± 280.527**	**28**	36.49 ± 5.545	62.44 ± 14.987 ^a^
	**Base gel**	**0.30 ± 0.040** ^**b**^	**582.07 ± 22.30** ^**a**^		26.78 ± 1.728	27.93 ± 0.861 ^b^
	**Gengigel ^®^**	**0.09 ± 0.044** ^**a**^	**1082.87 ± 50.217** ^**b**^		34.42 ± 19.269	35.93 ± 20.740

The parameters examined to show the effects of each treatment were the residual socket value (RSV) in the clinical observation and the number of fibroblasts, osteoblasts, and osteocytes in the histopathological observation. The values were shown as average ± standard deviation of at least three replicates. Different alphabets indicated significant statistical differences (p < 0.05) between the treatments analysed with the LSD test. The lack of alphabets or the same alphabets indicated no statistical differences (p > 0.05) between the treatments analysed with the LSD test.

### Histopathological analysis

**Figure 4.**
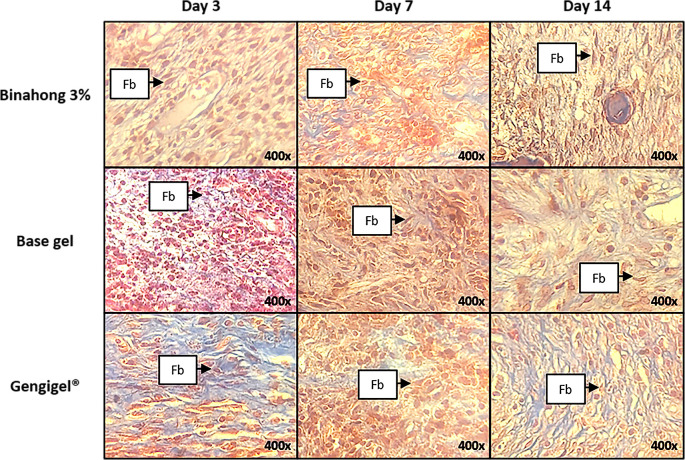
Histopathological observation of fibroblasts in the alveolar bone healing of sockets. The observation was conducted on the 3 ^rd^, 7 ^th^, and 14 ^th^ day post-extraction socket wounds stained with Masson’s Trichrome. (Abbreviation: Fb: Fibroblasts (×400)).

Fibroblast proliferation was examined on the 3 ^rd^, 7 ^th^, and 14 ^th^ day post-extraction. There was an increase trend in the number of fibroblasts from the 3 ^rd^ to 7 ^th^ day and a decrease from the 7 ^th^ to 14 ^th^ day ( Figure 3B). Fibroblasts can be seen among the blue-stained collagen fibre ( Figure 4). Based on the one-way ANOVA test, there were significant differences in the number of fibroblasts on the 3 ^rd^, 7 ^th^, and 14 ^th^ day in the Binahong, Gengigel ^®^, and base gel groups (p < 0.05). In the Binahong group, there were significant differences in the number of fibroblasts between the 3 ^rd^ and 7 ^th^ day and between the 7 ^th^ and 14 ^th^ day, while in the Gengigel ^®^ group, there were significant differences in the number of fibroblasts between the 3 ^rd^ and 7 ^th^ day and between the 3 ^rd^ and 14 ^th^ day. In the base gel group, the significant differences were only observed between the 3 ^rd^ and 7 ^th^ day ( Table 1).

Significant differences in the number of fibroblasts among the Binahong group, Gengigel ^®^ group, and the base gel group were observed on the 3 ^rd^, 7 ^th^, and 14 ^th^ day (p < 0.05). On the 3 ^rd^ day, there were significant differences in the number of fibroblasts between the Binahong group and the base gel group and between the Gengigel ^®^ group and the base gel group. On the 7 ^th^ day, there were significant differences between the Binahong group and the Gengigel ^®^ group, between the Binahong group and the base gel group, and between the Gengigel ^®^ group and the base gel group. On the 14 ^th^ day, a significant difference was only observed between the Gengigel ^®^ group and the base gel group ( Table 2).

**Figure 5.**
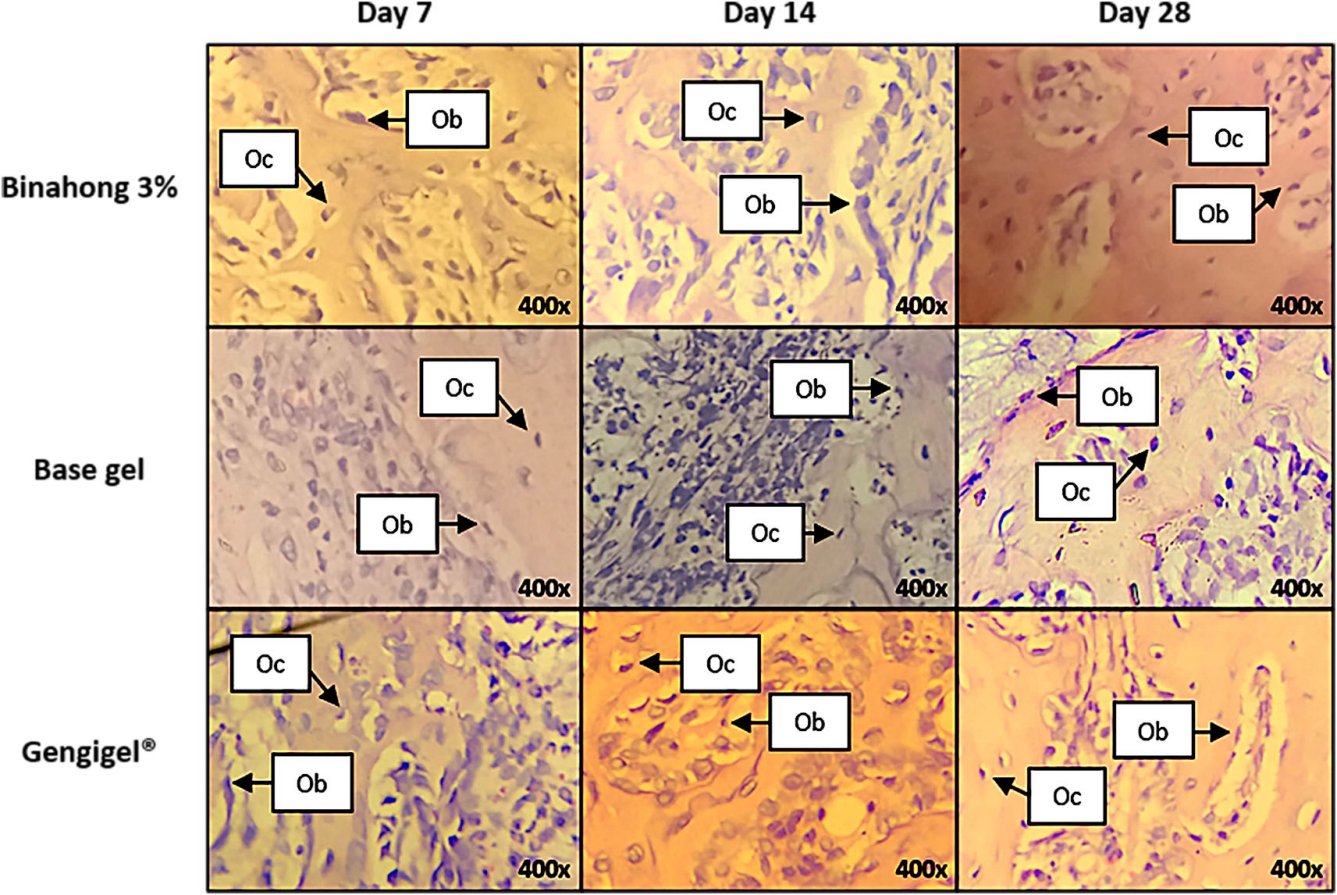
Histopathological observation of osteoblasts and osteocytes in the alveolar bone healing of sockets. The observation was conducted on the 7 ^th^, 14 ^th^, and 28 ^th^ day post-extraction socket wounds stained with haematoxylin-eosin. (Abbreviations: Ob: osteoblasts; Oc: osteocytes (×400)).

The proliferation of osteoblasts was examined on the 7 ^th^, 14 ^th^, and 28 ^th^ day post-extraction. In the Binahong group and the Gengigel ^®^ group, there was an increase trend in the number of osteoblasts from the 7 ^th^ to 14 ^th^ day and a decrease from the 14 ^th^ to 28 ^th^ day, although in the base gel group, the number of osteoblasts kept rising over time ( Figure 3C). Osteoblasts can be seen along the alveolar bone matrix ( Figure 5). Based on the one-way ANOVA test, there were significant differences in the number of osteoblasts among the 7 ^th^, 14 ^th^, and 28 ^th^ day in the Binahong group and the base gel group (p < 0.05), while the Gengigel ^®^ group showed no differences in the number of osteoblasts among those days. In the Binahong group, there were significant differences between the 7 ^th^ and 14 ^th^ day and between the 7 ^th^ and 28 ^th^ day, while in the base gel group, there were significant differences between the 7 ^th^ and 14 ^th^ day, between the 7 ^th^ and 28 ^th^ day, and between the 14 ^th^ and 28 ^th^ day ( Table 1).

The differences in the number of osteoblasts among the Binahong group, Gengigel ^® ^group, and the base gel group were only observed on the 14 ^th^ day post-extraction (p < 0.05). On the 14 ^th^ day, significant differences were observed between the Binahong group and the base gel group and between the Gengigel ^®^ group and the base gel group ( Table 2).

Osteocyte proliferation was examined on the 7 ^th^, 14 ^th^, and 28 ^th^ day post-extraction. In the Binahong group and the base gel group, there was a trend of increase in the number of osteocytes over time, although in the Gengigel ^®^ group, the increase only happened from the 7 ^th^ to 14 ^th^ day and was followed by a decrease from the 14 ^th^ to 28 ^th^ day ( Figure 3D). Osteocytes can be seen in the alveolar bone matrix inside lacunae ( Figure 5). Based on the one-way ANOVA test, there were significant differences in the number of osteocytes among the 7 ^th^, 14 ^th^, and 28 ^th^ day post-extraction only in the Binahong group and the base gel group (p < 0.05). No difference was observed in the Gengigel ^®^ group among those days. In the Binahong group, there were significant differences between the 7 ^th^ and 14 ^th^ day and between the 7 ^th^ and 28 ^th^ day, while in the base gel group, significant differences were observed between the 7 ^th^ and 14 ^th^ day, between the 7 ^th^ and 28 ^th^ day, and between the 14 ^th^ and 28 ^th^ day ( Table 1).

Differences in the number of osteocytes among the treatment groups were only observed on the 14 ^th^ and 28 ^th^ day post-extraction (p < 0.05). On the 14 ^th^ day, there were significant differences between the Binahong group and the base gel group and between the Gengigel ^®^ group and the base gel group. On the 28 ^th^ day, significant differences were observed only between the Binahong group and the base gel group ( Table 2).

## Discussion

The concentration of the Binahong leaf extract gel in this study was chosen based on a previous research by Hanafiah *et al*, which examined the effects of Binahong leaf extract gel on the healing of palatal mucosal wounds. ^30^ Among the various Binahong leaf extract gel concentrations in the research, 3% Binahong leaf extract gel showed the best affinity in increasing fibroblast proliferation. ^30^ In this study, the RSV in every treatment group decreased over time and the Binahong group showed a lower RSV in comparison with the other treatment groups on the 7 ^th^ and the 14 ^th^ day (p < 0.05). A lower RSV implied a decrease in socket wound size, so it can be assumed that the Binahong group showed a better affinity in accelerating wound closure compared to the positive and negative control groups. Fibroblast is one of the cells that affects wound retraction and it can be assumed that the acceleration of wound socket closure is influenced by the number of fibroblasts. ^35^

Aside from the ones in Gengigel® group, fibroblasts in this study increased from the 3 ^rd^ to the 7 ^th^ day and decreased from the 7 ^th^ to the 14 ^th^ day. According to Vieira *et al*, who examined the physiologic socket wound healing in mice, the fibroblasts in the alveolar bone would proliferate and would continue to increase until the 7 ^th^ day post-extraction. The number of fibroblasts would decrease from the 7 ^th^ day to the 14 ^th^ day following the formation of new alveolar bone. ^8^ On the 7 ^th^ day, the highest fibroblast proliferation was in the Binahong group. In the Gengigel ^®^ group, there was no significant difference in the number of fibroblasts between the 7 ^th^ day and the 14 ^th^ day because the hyaluronic acid in Gengigel ^®^ had more effects in the early phase of wound healing in fibroblast proliferation compared to alveolar bone formation. ^41^ Fibroblast can secrete collagen and extracellular matrix, which will make up the granulation tissue. Granulation tissue will then be replaced by the provisional matrix, which has fewer inflammatory cells and more matrix and new blood vessels. ^42^

In this study, osteoblasts increased from the 7 ^th^ day to the 14 ^th^ day and decreased from the 14 ^th^ day to the 28 ^th^ day. Osteoblast proliferation reached its peak on the 14 ^th^ day and started to decrease until the 28 ^th^ day, in which the osteoblasts had matured. ^8^ In the base gel group, osteoblast proliferation continued to increase until the 28 ^th^ day. It could be assumed that in the base gel group, the socket wound healing was still on the early phase of bone formation, which was shown by the high number of undifferentiated osteoblasts. Osteoblasts will produce a matrix, which is called osteoid, and osteoid will be mineralized, forming woven bone. ^42^ Osteoblasts that become trapped in the matrix will become osteocytes. Osteocytes have a role in managing bone remodelling because they can influence the activities of osteoblasts and osteoclasts. ^4^ In the study, there was an increase in the number of osteocytes from the 7 ^th^ day until the 28 ^th^ day in the Binahong group and the highest osteocyte number was on the 28 ^th^ day. The osteocytes were found among the alveolar bone matrix. This finding concurred with the experiment of Olaitan *et al* that showed a higher osteocyte proliferation in the 4 ^th^ week compared to the 2 ^nd^ week post-extraction. ^13^

Several studies on the toxicity of *Anredera cordifolia* leaf extract have been carried out. Research conducted by Salasanti CD, *et al.* proved that *Anredera cordifolia* ethanolic leaf extract did not cause mortality in rats. ^43^ The study showed no significant changes in body weight formation, organ weight, hematology, or blood biochemistry in rats. ^43^ Another study conducted by Sukandar EY, *et al.* showed that *Anredera cordifolia* ethanolic leaf extract could be administered up to 1000 mg/kg bw with no teratogenic effect. ^44^

The Binahong plant contains various secondary metabolites that function in the proliferation of fibroblasts, osteoblasts, and osteocytes, optimising wound healing and in turn lowering the value of RSV. In the Binahong leaf extract, there is saponin, which can increase the expression of TGF-α (transforming growth factor-alpha) and TGF-β (transforming growth factor-beta). ^19^^,^^45^ TGF-β can activate fibroblasts and TGF-α can activate Osterix, which will function in osteoblast differentiation. ^19^^,^^45^ Saponin is also antiseptic and it can affect cell membrane integrity and cause the lysis of pathogens, especially fungi. ^46^

Apigenin, a flavonoid found in Binahong, can increase the expression of TGF-β and PDGF (platelet-derived growth factor) that also function in fibroblast activation so that it can migrate towards the clot. ^47^^,^^48^ PDGF is also a protein that affects fibroblast proliferation. ^48^ Apigenin also has anti-inflammatory properties because it can inhibit the activation of NF-κB (nuclear factor kappa B). ^49^ Inhibited NF-κB can prevent the production of inflammatory mediators that can increase inflammation. ^49^ The other flavonoids in the Binahong plant that function in socket wound healing are quercetin and vitexin. ^50^^–^^52^ Quercetin has the ability to reduce osteoclast formation through inhibiting IL-17 (interleukin-17), which is induced by RANKL (receptor activator of nuclear factor kappa-B ligand), and quercetin can also increase osteogenesis, angiogenesis, and function as an antioxidant. ^50^^,^^51^ Vitexin can affect bone formation by increasing osteoblast differentiation through p-Smad (phosphorylation-small mother againts) and Runx2 (runt-related transcription factor 2). ^52^

The tannin in the Binahong plant functions in fibroblast migration because it can increase VEGF (vascular endothelial growth factor) in the early phase of wound healing. ^53^ VEGF is a protein that plays a role in fibroblast migration. ^48^ Tannin is also an antioxidant. Antioxidant is needed to neutralise free radicals that are produced during wound healing. Free radicals can damage cell protein structure, which will prevent cell proliferation. ^54^

The triterpenes in the Binahong leaf extract, such as ursolic acid and oleanolic acid, have anti-inflammatory, antiseptic, and antioxidant properties. ^19^^,^^55^ Ursolic acid can also influence the differentiation and proliferation of osteoblasts and improve the activity and mineralisation of ALP (alkaline phosphatase). ^56^

Previous studies have shown the antimicrobial and antiinflammatory activities in Binahong leaves in relation to its effectivity in some fields in dentistry, such as periodontal therapy, but this study has further proved that Binahong leaves have the potential to be utilised in other fields in dentistry, especially in terms of wound healing acceleration after surgical procedures. ^24^^,^^57^^,^^58^ More effort should be put in investigating the mechanism of how the plant works in healing injured tissue. The limitation of this study was that it simply showed the effects on 3% Binahong leaf extract gel on the proliferation of fibroblasts, osteoblasts, and osteocytes without studying the mechanism of how the substance could affect the proliferation. The 3% Binahong leaf extract gel used in this study also had to be constantly reproduced to ensure its freshness, and so there might be variability between the batches, despite the same composition. Future studies can be focused on the effects of 3% Binahong leaf extract gel on the proteins involved in fibroblast, osteoblast, and osteocyte proliferation. Further experiments can also be done to obtain a more stable and durable 3% Binahong leaf extract gel formulation or even to create a nanogel formulation as a more effective mode of delivery. The substance's effectiveness should also be tested on humans in clinical trials.

## Conclusion

The study concluded that the application of 3% Binahong leaf extract gel could enhance alveolar bone healing, which could be shown through the decreasing value of residual socket volume and the increasing proliferation of fibroblasts, osteoblasts, and osteocytes.

## Data availability

### Underlying data

Zenodo: The dataset of ‘The effects of 3% Binahong ( *Anredera cordifolia*) leaf extract gel on the alveolar bone healing in post-extraction tooth socket wound in Wistar rats ( *Rattus norvegicus*)’. https://doi.org/10.5281/zenodo.5189362. ^59^

This project contains the following underlying data:

• Raw data of fibroblast mean cell count.csv (Raw data of fibroblast mean cell count)
• Raw data of osteoblast mean cell count.csv (Raw data of osteoblast mean cell count)
• Raw data of osteocyte mean cell count.csv (Raw data of osteocyte mean cell count)
• Raw data of residual socket volume.csv (Raw data of residual socket volume)
• Readme.txt (Explanations about the raw data in the dataset)

Zenodo: Rat tooth extraction and 3% Binahong ( *Anredera cordifolia* (Ten.) STEENIS) leaf extract gel. https://doi.org/10.5281/zenodo.5202954. ^60^

This project contains the following underlying data:

• Rat anesthesia.tiff (Intraperitoneal anesthesia on the rat)
• Rat tooth extraction.tiff (The extraction of the rat’s mandibular left incisor)
• The Binahong leaf extract gel.tif (3% Binahong leaf extract gel)
• The Binahong leaf extract.tif (Binahong leaf extract)

### Reporting guidelines

Zenodo: ARRIVE checklist for ‘The effects of 3% Binahong ( *Anredera cordifolia*) leaf extract gel on the alveolar bone healing in post-extraction tooth socket wound in Wistar rats ( *Rattus norvegicus*)'. https://doi.org/10.5281/zenodo.5203068. ^61^

Data are available under the terms of the Creative Commons Attribution 4.0 International license (CC-BY 4.0).
