# Supplementary material for: Effects of 3% binahong ( Anredera cordifolia) leaf extract gel on alveolar bone healing in post-extraction tooth socket wound in Wistar rats ( Rattus norvegicus)
Source: F1000Res. 2022 Apr 5;10:923. Originally published 2021 Sep 15. [Version 2] doi: 10.12688/f1000research.72982.2 (PMC9024130; doi:10.12688/f1000research.72982.2)
Supplement: Peer review history

# Peer review: Reviewer response for version 2

**DOI:** 10.5256/f1000research.123005.r129877

**Published:** 2022-06-14

**Authors:** Lidia Audrey Rocha Valadas, Francineudo Oliveira Chagas

**Affiliations:** 1 Department of Preventive and Community Dentistry, University of Buenos Aires, Buenos Aires, Argentina; 2 Federal University of Ceará, Fortaleza, Brazil

We have checked that the authors followed my suggestions. We suggest Approved

Is the work clearly and accurately presented and does it cite the current literature?

Yes

If applicable, is the statistical analysis and its interpretation appropriate?

Yes

Are all the source data underlying the results available to ensure full reproducibility?

Yes

Is the study design appropriate and is the work technically sound?

Yes

Are the conclusions drawn adequately supported by the results?

Yes

Are sufficient details of methods and analysis provided to allow replication by others?

Yes

Reviewer Expertise:

Dentistry, Pharmacology; Biofilms; Natural products

We confirm that we have read this submission and believe that we have an appropriate level of expertise to confirm that it is of an acceptable scientific standard.

# Peer review: Reviewer response for version 2

**DOI:** 10.5256/f1000research.123005.r129875

**Published:** 2022-04-21

**Authors:** Mohd Farhan Hanif Reduan

**Affiliations:** 1 Department of Clinical Studies, Faculty of Veterinary Medicine, Universiti Malaysia Kelantan, Kelantan, Malaysia

I have checked the revised manuscript and found that the authors have addressed all the comments and the revisions are appropriate.

Is the work clearly and accurately presented and does it cite the current literature?

Yes

If applicable, is the statistical analysis and its interpretation appropriate?

Yes

Are all the source data underlying the results available to ensure full reproducibility?

Yes

Is the study design appropriate and is the work technically sound?

Yes

Are the conclusions drawn adequately supported by the results?

Yes

Are sufficient details of methods and analysis provided to allow replication by others?

Yes

Reviewer Expertise:

NA

I confirm that I have read this submission and believe that I have an appropriate level of expertise to confirm that it is of an acceptable scientific standard.

# Peer review: Reviewer response for version 1

**DOI:** 10.5256/f1000research.76599.r96680

**Published:** 2022-01-17

**Authors:** Mohd Farhan Hanif Reduan

**Affiliations:** 1 Department of Clinical Studies, Faculty of Veterinary Medicine, Universiti Malaysia Kelantan, Kelantan, Malaysia

The manuscript and the study is worth for indexing on this platform as it highlights the use of local plants available for the alveolar bone healing.

 Few considerations should be taken to improve the quality of the article:

1. Introduction - to include all pharmacological activities of the plant
2. Methodology - to cite for the H&E and Masson Trichome procedures. It is good if the authors can explain how to differentiate the various type of cells; fibroblasts, osteocyctes, osteoclasts and osteoblasts
3. Results - the good quality of histopathology figures must be included in the article, referring to Figure 2: the bluish stain fibroblasts cannot be seen clearly by the reviewers and readers. Same comment for the Figure 3.
4. Discussion - to include future direction of the present study, the limitation of the current study can be added and explained.
5. Conclusion - Well concluded

Is the work clearly and accurately presented and does it cite the current literature?

Yes

If applicable, is the statistical analysis and its interpretation appropriate?

Yes

Are all the source data underlying the results available to ensure full reproducibility?

Yes

Is the study design appropriate and is the work technically sound?

Yes

Are the conclusions drawn adequately supported by the results?

Yes

Are sufficient details of methods and analysis provided to allow replication by others?

Yes

Reviewer Expertise:

Histopathology, natural product development, animal study, veterinary

I confirm that I have read this submission and believe that I have an appropriate level of expertise to confirm that it is of an acceptable scientific standard, however I have significant reservations, as outlined above.

# Response

**Published:** 2022-03-29

**Authors:** Olivia Avriyanti Hanafiah

**Affiliations:** University of Sumatera Utara, Indonesia

We thank the reviewer for reviewing our manuscript and offering their suggestions. Please find below a point-by-point response to the comments. We hope that the reviewer would find the modifications satisfactory.

**Comment:** Introduction - to include all pharmacological activities of the plant

**Response:** Thank you for the insight, we have added this in Paragraph 4 of Introduction.

**Comment: **Methodology - to cite for the H&E and Masson Trichome procedures. It is good if the authors can explain how to differentiate the various type of cells; fibroblasts, osteocyctes,

 osteoclasts and osteoblasts

**Response:** Citations have been added in the "Histopathological analysis" section in Methods. Details about cells have also been added in the same section. Thank you for the input.

**Comment:** Results - the good quality of histopathology figures must be included in the article, referring to Figure 2: the bluish stain fibroblasts cannot be seen clearly by the reviewers and readers. Same comment for the Figure 3.

**Response:** Following the suggestion, we have uploaded improved versions of Figure 2 and Figure 3 (Now referred to as Figure 4 and Figure 5 in the new version)

**Comment: **Discussion - to include future direction of the present study, the limitation of the current study can be added and explained.

**Response: **Following the suggestions, we have added these details in the last paragraph in Discussion.

**Comment:** Conclusion - Well concluded

**Response: **We thank you for this input.

# Peer review: Reviewer response for version 1

**DOI:** 10.5256/f1000research.76599.r101384

**Published:** 2022-01-17

**Authors:** Dalia Hussein El-Rouby

**Affiliations:** 1 Department of Oral and Maxillofacial Pathology, Faculty of Dentistry, Cairo University, Giza, Egypt

**Effects of 3% binahong ( *Anredera cordifolia*) leaf extract gel on alveolar bone healing in post-extraction tooth socket wound in Wistar rats ( *Rattus norvegicus*)**

 The points that should be addressed to make the article scientifically sound:

**Introduction:**

 'The healing of soft tissue, which are comprised of the gingiva and connective tissue...' - Please revise the language style

 Binahong: the letter B should always be capitalised

**Methods:**

 Sample size calculation: 48 male Wistar rats were randomly allocated to twelve groups. Please mention the effect size obtained from the previous study (Khoswanto et al, 2019)

 Rats with abnormalities or those that died before the experiment ended were excluded from the study: how did you replace rats who died before the end of the study to maintain the sample size?

**Results**

 Please provide figures showing the 95% confidence interval in error bars

 The residual socket volume (RSV): Please specify the unit of measurement (mm ^3^ ?) in the graph, table 2 and text

Is the work clearly and accurately presented and does it cite the current literature?

Yes

If applicable, is the statistical analysis and its interpretation appropriate?

Yes

Are all the source data underlying the results available to ensure full reproducibility?

No source data required

Is the study design appropriate and is the work technically sound?

Yes

Are the conclusions drawn adequately supported by the results?

Yes

Are sufficient details of methods and analysis provided to allow replication by others?

Yes

Reviewer Expertise:

Oral tumors, use of natural products for chemo-prevention and improving oral condition, use of bone substitutes

I confirm that I have read this submission and believe that I have an appropriate level of expertise to confirm that it is of an acceptable scientific standard, however I have significant reservations, as outlined above.

# Response

**Published:** 2022-03-29

**Authors:** Olivia Avriyanti Hanafiah

**Affiliations:** University of Sumatera Utara, Indonesia

We thank the reviewer for reviewing our manuscript and offering their suggestions. Please find below a point-by-point response to the comments. We hope that the reviewer would find the modifications satisfactory.

**Comment:** 'The healing of soft tissue, which are comprised of the gingiva and connective tissue...' - Please revise the language style

**Response: **Thank you for the suggestion, we have revised the diction of this sentence.

**Comment: **Binahong: the letter B should always be capitalised

**Response:** Thank you for the insight, we have thus made the changes.

**Comment:** Sample size calculation: 48 male Wistar rats were randomly allocated to twelve groups. Please mention the effect size obtained from the previous study (Khoswanto et al, 2019)

**Response: **We have added this detail in "Sample Size" section in Methods, thank you.

**Comment:** Rats with abnormalities or those that died before the experiment ended were excluded from the study: how did you replace rats who died before the end of the study to maintain the sample size?

**Response: **The rats were not replaced as long as the minimal sample size were still maintained. Elaboration on this can be found in "Animals" section in Methods.

**Comment:** Please provide figures showing the 95% confidence interval in error bars

**Response:** Following the suggestion, we have added these details in the improved Figure 3.

**Comment:** The residual socket volume (RSV): Please specify the unit of measurement (mm3 ?) in the graph, table 2 and text

**Response:** Thank you for the suggestion. We have thus added the unit of measurement in the graph, table, and text for fibroblast, osteoblast, and osteocyte proliferation. Unfortunately, there is no unit of measurement for RSV as it is the ratio of end volume and beginning volume (end volume: beginning volume).

# Peer review: Reviewer response for version 1

**DOI:** 10.5256/f1000research.76599.r96681

**Published:** 2021-11-03

**Authors:** Lidia Audrey Rocha Valadas

**Affiliations:** 1 Department of Preventive and Community Dentistry, University of Buenos Aires, Buenos Aires, Argentina

This research is an experimental study on the use of *Anredera cordifolia* in gel form to be used after extraction.

 The study is innovative especially in the area, with few works in the literature about it. In addition, the methodology has reproducibility.

**A) Introduction:**

 1 - I suggest a paragraph about the medicinal properties reported in the literature and not just isolated constituents.

 2 - There was a gap between the 2nd and 3rd paragraphs, before starting the third, I should start by addressing the use of medicinal plants for this purpose.

 3 - I suggest checking the articles of these authors, it may help (some are from medicinal plants): Alexandre *et al.* 2018 ^1^, Guimarães *et al.* 2016 ^2^, Lima MDR *et al.* 2017 ^3^, Lima V *et al.* 2020 ^4^, Ribeiro *et al.* 2018 ^5^, and Teixeira *et al.* 2017 ^6^.

**B) Methodology:**

 4 - If it is a study with natural products, I suggest improving the details of georeferencing, with the addition of coordinates and season.

 5 - I suggest a flowchart for the division of groups and a chart with the constituents of the gel.

**C) Discussion:**

 6 - In the discussion, I suggest reviewing the second and third paragraphs so as not to repeat information that is already in the results.

 7 - I also believe it is important to talk about the cytotoxicity and safety of the species, since the intention is that the gel will one day be clinically applicable.

 8 - To further address the importance of investigating properties of natural products in Dentistry, especially in surgery, since most studies are related to cariogenic or periodontal biofilm.

 9 - Finally, add limitations and future perspectives.

Is the work clearly and accurately presented and does it cite the current literature?

Yes

If applicable, is the statistical analysis and its interpretation appropriate?

Yes

Are all the source data underlying the results available to ensure full reproducibility?

Yes

Is the study design appropriate and is the work technically sound?

Yes

Are the conclusions drawn adequately supported by the results?

Yes

Are sufficient details of methods and analysis provided to allow replication by others?

Yes

Reviewer Expertise:

Dentistry, Pharmacology; Biofilms; Natural products

I confirm that I have read this submission and believe that I have an appropriate level of expertise to confirm that it is of an acceptable scientific standard, however I have significant reservations, as outlined above.

## References

[1] : Anti-inflammatory and antiresorptive effects of Calendula officinalis on inflammatory bone loss in rats. *Clin Oral Investig* .2018;22(6) : 10.1007/s00784-017-2308-7 2175-2185 10.1007/s00784-017-2308-7 29288400

[2] : Dry Extract of Matricaria recutita L. (Chamomile) Prevents Ligature-Induced Alveolar Bone Resorption in Rats via Inhibition of Tumor Necrosis Factor-α and Interleukin-1β. *J Periodontol* .2016;87(6) : 10.1902/jop.2016.150411 706-15 10.1902/jop.2016.150411 26777767

[3] : The Effect of Calendula officinalis on Oxidative Stress and Bone Loss in Experimental Periodontitis. *Front Physiol* .2017;8: 10.3389/fphys.2017.00440 440 10.3389/fphys.2017.00440 28701962PMC5487466

[4] : Uncaria tomentosa reduces osteoclastic bone loss in vivo. *Phytomedicine* .2020;79: 10.1016/j.phymed.2020.153327 153327 10.1016/j.phymed.2020.153327 32920290

[5] : Tocoyena sellowiana extract decreases bone loss in an experimental model of periodontitis in rats: Putative role for cyclooxygenase-2 and IL-1? inhibition. *Biomed Pharmacother* .2018;98: 10.1016/j.biopha.2018.01.011 863-872 10.1016/j.biopha.2018.01.011 29571257

[6] : Stemodia maritima L. Extract Decreases Inflammation, Oxidative Stress, and Alveolar Bone Loss in an Experimental Periodontitis Rat Model. *Front Physiol* .2017;8: 10.3389/fphys.2017.00988 988 10.3389/fphys.2017.00988 29249988PMC5717945

# Response

**Published:** 2022-03-29

**Authors:** Olivia Avriyanti Hanafiah

**Affiliations:** University of Sumatera Utara, Indonesia

We thank the reviewer for reviewing our manuscript and offering their suggestions. Please find below a point-by-point response to the comments. We hope that the reviewer would find the modifications satisfactory.

**Comment:** I suggest a paragraph about the medicinal properties reported in the literature and not just isolated constituents.

**Response:** We have elaborated on the medicinal properties of Binahong in paragraph three in Introduction.

**Comment:** There was a gap between the 2nd and 3rd paragraphs, before starting the third, I should start by addressing the use of medicinal plants for this purpose.

**Response:** We have followed this suggestion, thank you for the insight.

**Comment: **I suggest checking the articles of these authors, it may help (some are from medicinal plants): Alexandre et al. 20181, Guimarães et al. 20162, Lima MDR et al. 20173, Lima V et al. 20204, Ribeiro et al. 20185, and Teixeira et al. 20176.

**Response:** Thank you for the suggestions by the reviewer. We have read the articles and they have provided us with some helpful insights.

**Comment:** If it is a study with natural products, I suggest improving the details of georeferencing, with the addition of coordinates and season.

**Response:** We have added these details in "Binahong leaf extract gel and base gel preparation" section in Methods.

**Comment:** I suggest a flowchart for the division of groups and a chart with the constituents of the gel.

**Response:** Following the suggestion by the reviewer, we have added these details in Figure 1 and Figure 2.

**Comment:** In the discussion, I suggest reviewing the second and third paragraphs so as not to repeat information that is already in the results.

**Response:** Thank you for the suggestion, we have made some changes in paragraph two and three in Discussion.

**Comment:** I also believe it is important to talk about the cytotoxicity and safety of the species, since the intention is that the gel will one day be clinically applicable.

**Response: **Thank you for this insight, we have thus added information about the toxicity of Binahong, which can be found in the fourth paragraph in Discussion

**Comment:** To further address the importance of investigating properties of natural products in Dentistry, especially in surgery, since most studies are related to cariogenic or periodontal biofilm.

**Response: **As suggested by the reviewer, we have added this in the last paragraph in Discussion

**Comment:** Finally, add limitations and future perspectives.

**Response: **As suggested by the reviewer, we have added this in the last paragraph in Discussion
